# A new perspective in understanding rainfall from satellites over a complex topographic region of India

**DOI:** 10.1038/s41598-019-52075-y

**Published:** 2019-10-30

**Authors:** Manoj Kumar Thakur, T. V. Lakshmi Kumar, K. Koteswara Rao, Humberto Barbosa, V. Brahmananda Rao

**Affiliations:** 1 Atmospheric Science Research Laboratory, Department of Physics, SRM Institute of Science and Technology, Kattankulathur, 603203 India; 2 Tribhuvan University, Kathmandu, Nepal; 3 Centre for Climate Change Research, Indian Institute of Tropical Meteorology, Pune, India; 4 Laboratorio de Analise e Processamento de Imagens de Satelites, Universiadade Federal de Alogoas- UFAL, Maceió, Brazil; 5 Instituto Nacional de Pesquisas Espaciais, INPE C.P. 515, São José dos Campos, SP 12245-970 Brazil

**Keywords:** Atmospheric dynamics, Space physics

## Abstract

Present study focuses on rainfall over Western Ghats (WG), a complex topographic region (elevation > 500 m) of India to evaluate and to better understand the satellite behavior in contrast with a flat region (FR) (elevation < 500 m) of central India from 1998 to 2016 using the combinatory data sets of TMPA and IMERG (satellite rainfall estimation). The categorical Intra Seasonal Oscillations (ISO) of Indian summer monsoon (ISM) namely, Madden Julian Oscillation (MJO) and Quasi Bi-Weekly Oscillation (QBWO) are tested in satellite and India Meteorological Department (IMD) gridded rainfall data sets to find out the satellite performance. As the accurate estimation of rainfall from satellites over higher elevation zones is challenging, here we propose a new perspective to select the rainfall products of satellite for better comparison with ground measurements. Considering the satellite’s best capability in detecting the cold clouds resulting from deep convection and its coupling with higher-level circulation, we show that the rainfall from satellites yield fruitful comparison with ground measurements when moist static stability, tropical easterly jet is above the climatological values.

## Introduction

Rain gauge network over land is the most trusted instrument for accurate rainfall measurement. However, in remote areas (e.g. mountainous terrain) sparse rain gauges make the rainfall data erroneous when are averaged over a region. Hence, satellite rainfall retrieval is an alternate option for optimal spatio-temporal analysis of precipitation over such remote areas. In recent times, availability of passive microwave and space borne active precipitation radar along with Infrared technique revolutionized the rainfall estimation and its characteristic study from space^1^. TMPA [Tropical Rainfall Measuring Mission (TRMM) Multi-satellite Precipitation Analysis] and IMERG [Integrated Multi-satellitE Retrievals for Global Precipitation Measurement (GPM)] are the promising multi-satellite precipitation retrieval, which provide opportunities to explore more about weather and monsoon systems over tropical region. Huffman *et al*.^2,3^ provide detailed information on TMPA and IMERG gridded rainfall data including their retrieval algorithm. These data sets are ideal for monsoon and hydrological studies^4^ although they need calibration at different scales with ground truth obtained from gauge and radar. It is reported that satellite rainfall estimation (e.g. INSAT-3D, TMPA, CMORPH) shows regional, seasonal and year to year bias^5–11^. Gruber and Levizzani^12^, Kikuchi and Wang^13^ summarized, satellite retrieval of rainfall from Infrared, microwave and its combination shows serious limitations mainly over land than over ocean. Dinku *et al*.^14^ reported that high altitudinal regions pose unique challenges to satellite precipitation retrieval and found that the underestimation of rainfall from the satellite over mountain is due to their incapability in distinguishing the raining and non-raining clouds when IR technique is employed. In cases, where orographic rain is not due to ice aloft, since passive microwave rainfall retrievals are mainly from ice scattering at the upper parts of convective clouds. It also underestimates the surface rain over mountain. Ebert *et al*.^15^ also confirmed that satellite generally captures convective precipitation but has difficulty to detect warm rain. Underestimation of rainfall by the satellites over mountainous region has been studied^16–19^ in detail and reported that it is also due to the complex nature of the topography over the mountains. Mitra *et al*.^8^ made extensive study on rainfall estimation over WG and reported that daily TRMM rainfall underestimate the heavy orographic precipitation. However, a better understanding of satellite rainfall characteristics is important for its effective utilization during the natural hazards such as landslides, floods during active monsoon time. As the spatio-temporal resolution of the satellite data is available on finer scale, study of aforementioned hazards could be possible when the satellite data are used with care.

In the present investigation, we attempt to study rainfall behavior over a complex topographic region, WG using rainfall data of TMPA and IMERG from TRMM and GPM (a follow-on mission to TRMM) constellation of satellites respectively from 1998 to 2016. The contrast in rainfall behavior during southwest (SW) monsoon season (June to September) over the elevated region of WG with a flat region in central India is presented, using typical Intra Seasonal Oscillation (ISO) indices associated with Indian summer monsoon namely MJO (~30 to 60 days), TEJ (~14 days) and MSS (~14 days) with small periodicity between 2 to 6 days. IMD and satellite data sets were compared over the FR and WG with a perspective to bring them to close agreement in certain conditions where the satellite rainfall data sets with existing algorithm are better compared with IMD rainfall.

## Results and Discussion

**Figure 1:**
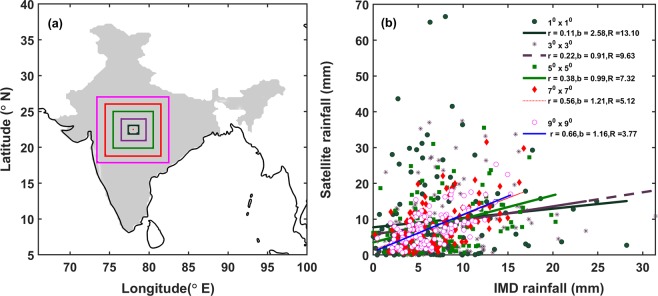
Region of spatial assessment over India (**a**) and distribution of IMD and IMERG rainfall at different spatial scale (**b**) [r (correlation), b (bias/mm), R (RMSE/mm)].

The spatial correspondence of newly released IMERG V5 and IMD is assessed by considering the different size grid boxes starting from 1° × 1° to 9° × 9° grids [Fig. 1(a)] from 2014–2016 during SW monsoon season. As the grid size increases from 1° to 9°, pearson correlation (r) between the two rainfall data sets increases from 0.11 to 0.66 [Fig. 1(b)] and mean daily bias is gradually decreased in 1° to 5° grid boxes from 2.58 mm to 0.99 mm, there after it increased in 7° × 7° and 9° × 9° boxes (1.21 and 1.16 mm respectively) though the correlation (r) is higher (0.56 and 0.66 respectively) over these regions. The observed increase in bias (by ~20%) might be due to the diverse topography comprising of flat and elevated regions where satellite behaves differently even in short distances^20,21^. This analysis has been done for understanding the agreement between IMERG and IMD rainfall data sets over India.

**Figure 2:**
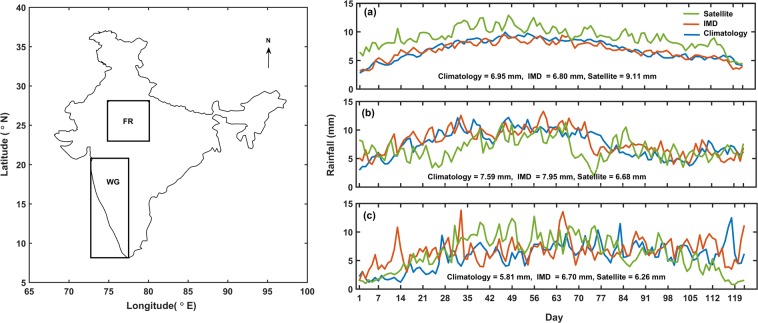
Study area (left panel) and Time series plot of daily rainfall (**a**) all India, (**b**) WG and (**c**). FR from 1998–2016 (right panel).

Prime aim of this study is to characterize rainfall from satellites over the complex topographic region, WG in order to use these data sets for research and application studies more reliably. For this purpose, we have selected a 5° × 5° box (23°N–28°N, 75°E–80°E) in central India region where the elevation is less than 500 m to examine the changes with respect to an elevated region greater than 500 m over WG [Fig. 2 left panel].

The daily time series of all India/WG/FR for 19-year (1998–2016) mean satellite retrieved rainfall, IMD rainfall and climatological IMD rainfall (1961–1990) during SW monsoon season are shown in [Fig. 2] right panel. Rainfall above 8 mm/day over WG is underestimated by satellite [Fig. 2b], as reported in the other studies over mountainous region of Iran as well as WG^22,23^. In order to verify the underestimation of daily satellite rainfall over the complex topographic region, a bootstrapped Kolnogorov-Smirnov test (hypothesis: greater) is performed to compare IMD and satellite daily rainfall over WG each year (1998–2016) during SW monsoon and it is found that the returned value of test statistics: h = 0 indicates that the K-S test accept the null hypothesis at the 5% significance level confirming underestimation of satellite rainfall over WG. An important requirement before the use of satellite data for verifying the rainfall characteristics is their uncertainties in the difference with the ground data^24^. If the bias is high, then it has to be assessed and evaluated. In the present study, the systematic bias of satellite rainfall with IMD rainfall over all India, WG and FR is 2.31, 1.27 and 0.44 mm/day respectively.

### Intra seasonal oscillation (ISO) of SW monsoon rainfall observed in IMD and satellite data sets

The time scale of ISO during monsoon season over India is 10 to 90 days and it has some preferred bands between 30 to 60 days, and less than 20 days^25–27^. Here we analyze the above-mentioned oscillations over all India, WG and FR separately, using Lomb Scargle Periodogram (LSP) a well-known algorithm for detecting periodic signals^28^.

**Figure 3:**
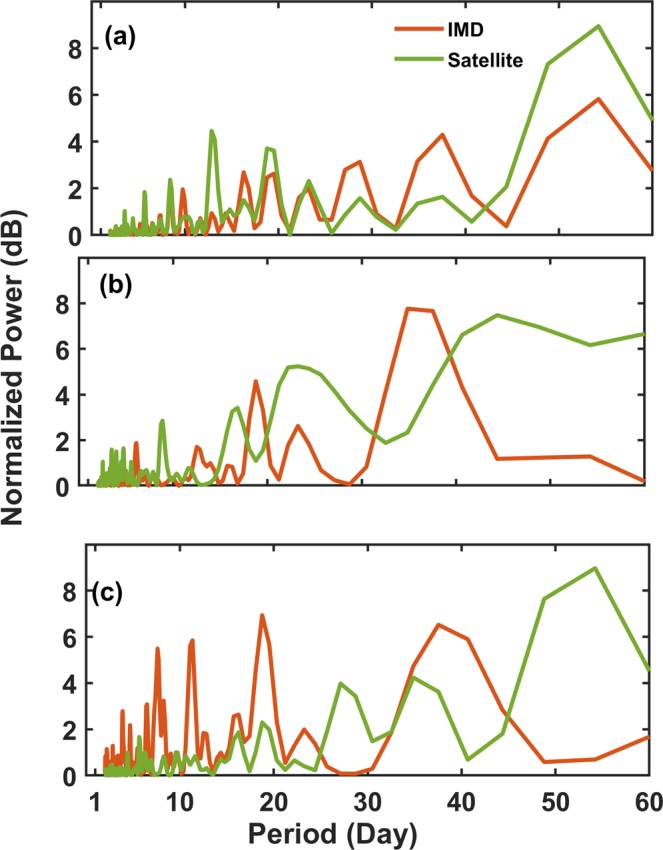
Lomb Scargle Periodogram (LSP) of IMD and satellite mean daily rainfall over (**a**) all India (**b**) WG and (**c**) FR during SW monsoon season from 1998–2016.

Figure 3(a–c) depicts LSP of IMD and satellite mean daily rainfall over all India [Fig. 3a], WG [Fig. 3b] and FR [Fig. 3c] respectively. From [Fig. 3], the preponderant features of ISO such as (MJO), and Quasi Bi-Weekly Oscillation (QBWO) are evident from the significant peaks observed between 30–60 days and less than 20 days time period respectively. The normalized power of rainfall during MJO (30–60 days period) is high in FR compared to that of WG [Fig. 3b]. This is in contrast to the IMD rainfall behavior where the normalized power of rainfall during MJO is low over FR and high over WG. In lower side of the 20 days period, significant peaks are observed in satellite rainfall normalized power over WG than that of FR where IMD rainfall normalized power is significant.

#### Madden julian oscillation (MJO)

Rainfall behavior of satellite datasets during MJO is evaluated, during the days when MJO cycle is in Indian Ocean i.e. in phase 2 and 3^29^. Pai *et al*.^30^ study shows when MJO cycle is in phase 2/phase 3, break/active events are most likely to occur over Indian region and hence we have selected aforementioned phases to consider both events of SW monsoon. MJO index $[{\rm{MJI}}=\sqrt{(RMM{1}^{2}+RMM{2}^{2})}]$ is used to distinguish weak MJO (MJI < 1) and strong MJO (MJI > 1) days for a year. Every year weak MJO days and strong MJO days when MJO is in phase 2 and 3 are identified during the SW monsoon season of 1998 to 2016 with maximum number of days in the year 2008 (43 days) and 2011 (31 days) when MJI > 1 and MJI < 1 respectively.

**Figure 4:**
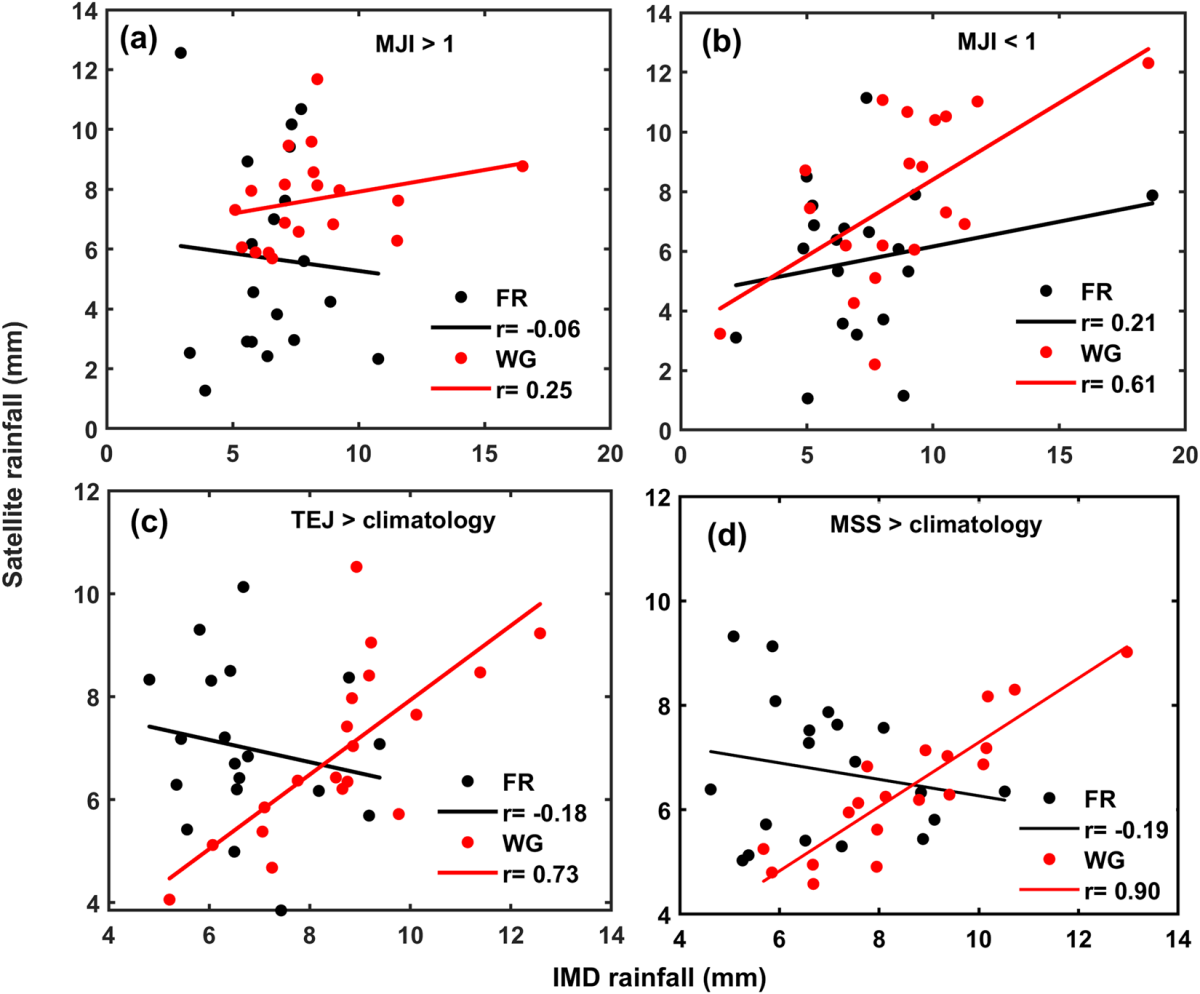
Scatter plot IMD rainfall and Satellite rainfall when (**a**) MJI > 1 (**b**) MJI < 1 (**c**) TEJ > climatology (**d**) MSS > climatology value over FR and WG.

By taking the satellite and IMD seasonal mean rainfall when MJI > 1 and MJI < 1 during the study period over FR and WG, correlations were obtained to understand the pattern and discrepancy between the two data sets. Satellite and IMD rainfall have shown good agreement over WG when MJI < 1, which is evidenced by significant correlation coefficient (r = +0.61) between the two data sets as compared with FR where the correlation is not statistically significant (r = +0.21) [Fig. 4(a–b)]. The estimation of rainfall from the satellite seems to be better over WG compared to FR when MJI > 1. The pearson correlation (r) between the two rainfall data sets over FR and WG are −0.06 and +0.25 respectively which are not significant. Further, we categorized the MJO with early periodicity (less than 40 days) and late periodicity (between 40–60 days) and carried out the same analysis. When the periodicity is greater than 40 days the correlation (r) between the two rainfall products is −0.25 over FR and +0.57 over WG. As the early appearance of MJO brings more rain showers and whereas late arrival of MJO causes deficient rainfall, our analysis shows that the linear association between the two data sets is significant (r = +0.57) over WG even in dry conditions.

#### Quasi Bi-weekly oscillation (QBWO)

Further, agreement between rainfall products of IMD and satellites during SW monsoon is assessed during QBWO. The elements of QBWO are: monsoon trough (~12 days period), mascarene high (~12 days period), TEJ (~14 days period), monsoon cloudiness(~14 days period), dry static stability(~15 days period) and moist static stability (~14 days period)^26^. In present study, we chose the elements of monsoon system TEJ and MSS as these two spectral components cover the period of QBWO (<20 days). TEJ is part of general circulation of upper troposphere and forms in the regions of Tibetan high and Equatorial Ocean, which produces rain based on its acceleration over Indian subcontinent. Being the large-scale circulation pattern, TEJ also interacts with orographic features and modify the rainfall^31^. MSS exhibits the stable and unstable influences during the active and break spells of Indian monsoon. TEJ, characterized by wind speed at 200 mb has been derived from reanalysis data for the study period over the respective WG and FR. Daily climatological (1998–2016) value of TEJ during SW monsoon season infers the strong winds over WG during the monsoon period. To study association between two rainfall data sets and their linkage to TEJ, daily satellite and IMD rainfall data have been picked every year during SW monsoon season from 1998–2016, when the TEJ of the day is greater than climatology value i.e. strong TEJ. When TEJ is above climatology, daily rainfall is averaged every year and correlation (r) is obtained between them. The scatter plots for the same are shown in [Fig. 4c].

The capability of satellite in estimating the rainfall over WG during above mentioned days is much better than that of FR, evidenced from significant correlation (r = +0.73) over WG and in contrast to FR (r = −0.18). As we have taken the criteria of TEJ > climatology, the present analysis infers significant relation of the two data sets during wet condition as higher TEJ corresponds to good monsoon activity. Strong TEJ allows the horizontal expansion of clouds that are forming due to the deep convection which favors the IR sensing of satellites to estimate the rainfall amounts more reliably^32^. Similar analysis has been done by taking the MSS over FR and WG. Ruscica *et al*.^33^ suggests that gradient of Moist Static Energy (MSE) i.e. MSS is important for the development of precipitation. When MSE increases, the vertical gradient of MSE between boundary layer and free atmosphere also increases, favoring unstable condition, which trigger precipitation^34^. MSS represent the mean atmospheric conditional instability over the Indian region during the monsoon season.

A high correlation (r = +0.90) is found between rainfall of IMD and satellite over WG during the day when MSS is greater than climatology. From the scatter plot [Fig. 4(d)], we can also observe that satellite failed to estimate the rainfall over FR which is evidenced by the insignificant correlation (r = −0.18). Higher MSE is due to increased evapotranspiration in an elevated moisture scenario, which enhances moist convection^35^. Studies^36^ show that the satellite algorithms are less sensitive to shallow convection compared to deep convective events leading to underestimation of rainfall. As in the satellite based rainfall estimates, IR based techniques dominate, empirical relationship between rainfall and cloud top temperature yield better results. For the deep convective clouds when the static stability is negative, the cloud top temperature is low and the clouds extend vertically and at higher altitudes, a better discrimination on the thresholds for convective and non-convective clouds makes the satellites to estimate the rainfall more accurately^36,37^. Since, WG is characterized by deep convective systems in the south Asian monsoon domain^38^ the association of satellite rainfall and IMD rainfall is better over WG compared to the FR of the present study. Further, the surface mean vertical velocity over WG and Flat regions (a 0.5° × 0.5° grid is selected in the two regions with co-ordinates 13.75°N to 14°N & 74.75°E to 75°E and 23.75°N to 24°N & 76.75°E to 77°E respectively) show deep convection over the WG evidenced by the higher mean surface vertical velocity (1.29 m/s over WG and −0.03 m/s over flat region for the SW monsoon period of 2004 to 2015 respectively) which is much higher than that of same in the grid of flat region (Supp. Fig. 1). Also, the Outgoing Longwave Radiation (OLR) values for the heavy rainfall events over the selected grid of WG and Flat region show lower values over the WG part compared to Flat part (Supp. Table 1). The lower OLR values are directly proportional to lower brightness temperatures of the clouds and indicate the deep convection/cold clouds^39,40^ which results in higher rainfall amounts. Deep convective clouds are the manifestation of the higher MSS and these clouds are better detected by the satellites (as discussed before). Over the flat region, lower mean surface vertical velocity and higher OLR results in shallow clouds, which are quite difficult to be detected by the satellites. However, the rainfall over WG is mainly contributed by the shallow clouds^41^, when monsoon currents are weak, the uncertainty in the comparison with IMD prevails. But, during the conditions of higher MSS/deep convective regimes, as it is the primary source for rainfall over WG^42^, the satellite shows better performance in estimating the rainfall and thus a good agreement persists with IMD data. Furthermore, the TEJ is known to be strongly connected to monsoon rainfall over the tropics^43^ and is linked to the monsoon circulation^44^. A strong TEJ is a characteristic feature of upper level monsoon circulation, it couples with the low level circulation and produces rainfall^45^. As the TEJ interacts with the low level monsoon current over the peninsular region of India^46^ (this will not cover FR considered in present study) satellite will be able to detect the clouds in a better way over the WG, thus providing the reliable estimates (when the TEJ is stronger i.e. above the climatology value).

**Figure 5:**
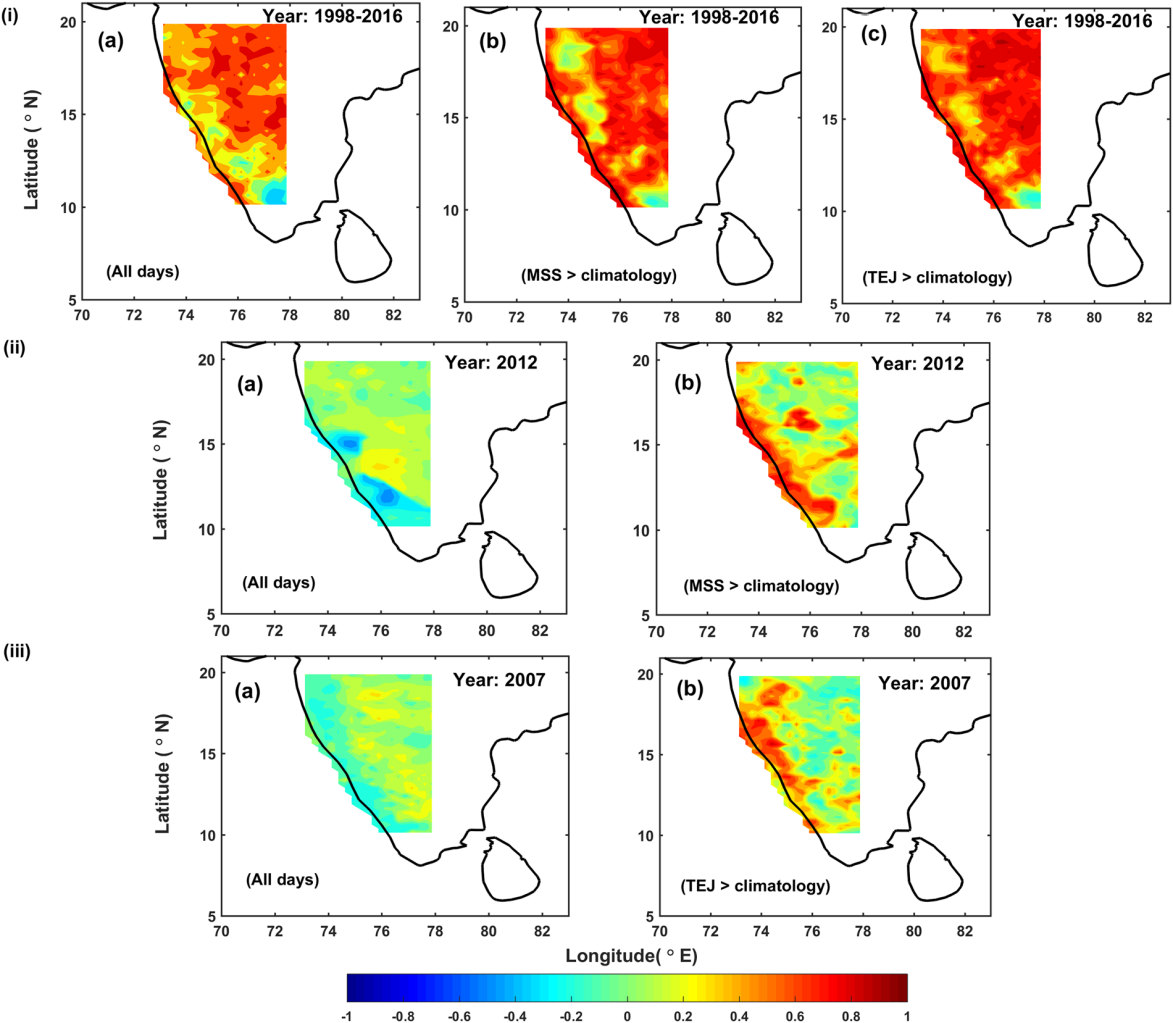
Spatial pattern of temporal correlation during SW monsoon (i) (**a**) for all 122 days, for the days when (**b**) MSS > climatology and (**c**) TEJ > climatology from 1998 to 2016,(ii) for the year 2012 same as (i) (**a,b**) (iii) for the year 2007 same as (i) (**a**,**c**).

**Figure 6:**
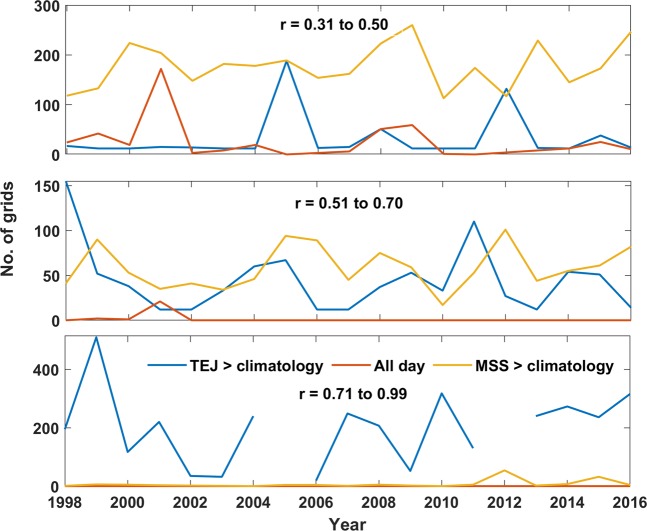
Number of grids in different correlation value (r) when TEJ/MSS > climatology and for all 122 days during SW monsoon.

[Figure 5(i)(a–c)] show the spatial pattern of temporal correlation over the WG between the daily rainfall data sets of IMD and satellite for all (19 × 122 = 2318) days of SW monsoon season of 19 years of study period and only for the days when MSS and TEJ are greater than their climatology (1368 and 1241 days respectively) for the same period respectively. [Fig. 5(i)] indicates the agreement between IMD and satellite does not considerably vary between the two cases. As the rainfall is averaged for a longer period (1998–2016), the frequency of rainfall fluctuations was smoothen out, so that there is no variation in the correlation. But, when the correlation pattern is considered for an individual year, the scenario is different. For example [Fig. 5(ii,iii)(a,b)] depict the same as [Fig. 5(i)], for the year 2012 (for all the SW monsoon season days(122) and the days when MSS > climatology) and 2007 (for all the SW monsoon season days(122) and the days when TEJ > climatology) respectively. In this case, the spatial pattern of temporal correlation varied substantially from [Fig. 5a(ii,iii)] to [Fig. 5b(ii,iii)]. An increase in correlation between two data sets, where the rainfall occurrence is high over WG, for example along the Malabar coast where the correlations show r = 0.60 to 0.99 when MSS is considered greater than climatology and similarly improved agreement is witnessed over the other parts of WG when TEJ is considered greater than climatology and which is not conspicuous when all the days were considered. Similar improved spatial association (high r -value) between two data sets is found every year from 1998 to 2016 when MSS/TEJ is considered greater than its climatology over WG. Increase in spatial coverage (number of grids) showing improved correlation with proposed criteria (MSS/TEJ > climatology) is presented in [Fig. 6].

Figure 6 shows number of grids in different correlations (r = 0.30 to 0.50; 0.51 to 0.70 and 0.71 to 0.99) between IMD and satellite rainfall for all 122 days (without criteria) and when MSS/TEJ is greater than climatology (with proposed criteria) from 1998–2016. The number of grids remarkably increased with the criterion (MSS/TEJ > climatology) over all the years of the study period. It can be seen that there is a partial agreement between the two data sets, which is evident by the correlation co-efficient when the daily data are considered without the criterion. The number of grids have been increased with a very good agreement indicated by the increasing number of grid points with higher correlations of 0.50 to 0.70 and 0.71 to 0.99 respectively when the proposed criteria is adopted [Fig. 6]. The average number of grids of the study period between IMD and satellite data sets when MSS and TEJ were considered greater than climatology is 200, 12 and 5 for the correlation (r) values ranging from 0.30 to 0.50; 0.51 to 0.70 and 0.71 to 0.99 respectively. The number of grids for the same correlation is 300, 100 and 55 with the proposed criterion i.e. during the days when TEJ and MSS greater than their climatology is taken into account. The overall analysis will be helpful to develop the reliable climate data sets of rainfall over WG using satellite estimates. As the SW monsoon has high spatial variability over India, the rainfall data sets over WG with the proposed criteria forms a new series of monsoon indices^47^.

## Conclusion

As most of the research reported earlier, the accuracy in satellite rainfall retrieval over mountainous terrain is a big challenge. An effort to assess the spatio-temporal scale at which the two datasets compare satisfactorily and achieved a criteria using Intra seasonal oscillation (ISO) over a terrain region, WG of India is made. This will help the users/policy makers to use the satellite data with more confidence. For this, TMPA 3B42 final and IMERG V5 final re-gridded daily rainfall products over WG and a selected FR in Central India from 1998 to 2016 have been used. Our analysis shows that Satellite daily rainfall can be used more reliably over the complex topographic region like Western Ghats of India during SW monsoon when TEJ/MSS over the region is greater than its climatology. Seasonal rainfall amount and daily precipitation spatial pattern show significant improvement in association between satellite retrieval and IMD rainfall. The satellite’s capability in providing the reliable estimate during the days of TEJ and MSS above the climatology is due to the coupling of low level circulation which arises due to the release of latent heat of cumulus convection with the upper level circulation of TEJ^48^. This enables the satellite to detect the lower thresholds of cloud top temperature and hence the better estimation.

## Data and Methods

**Figure 7:**
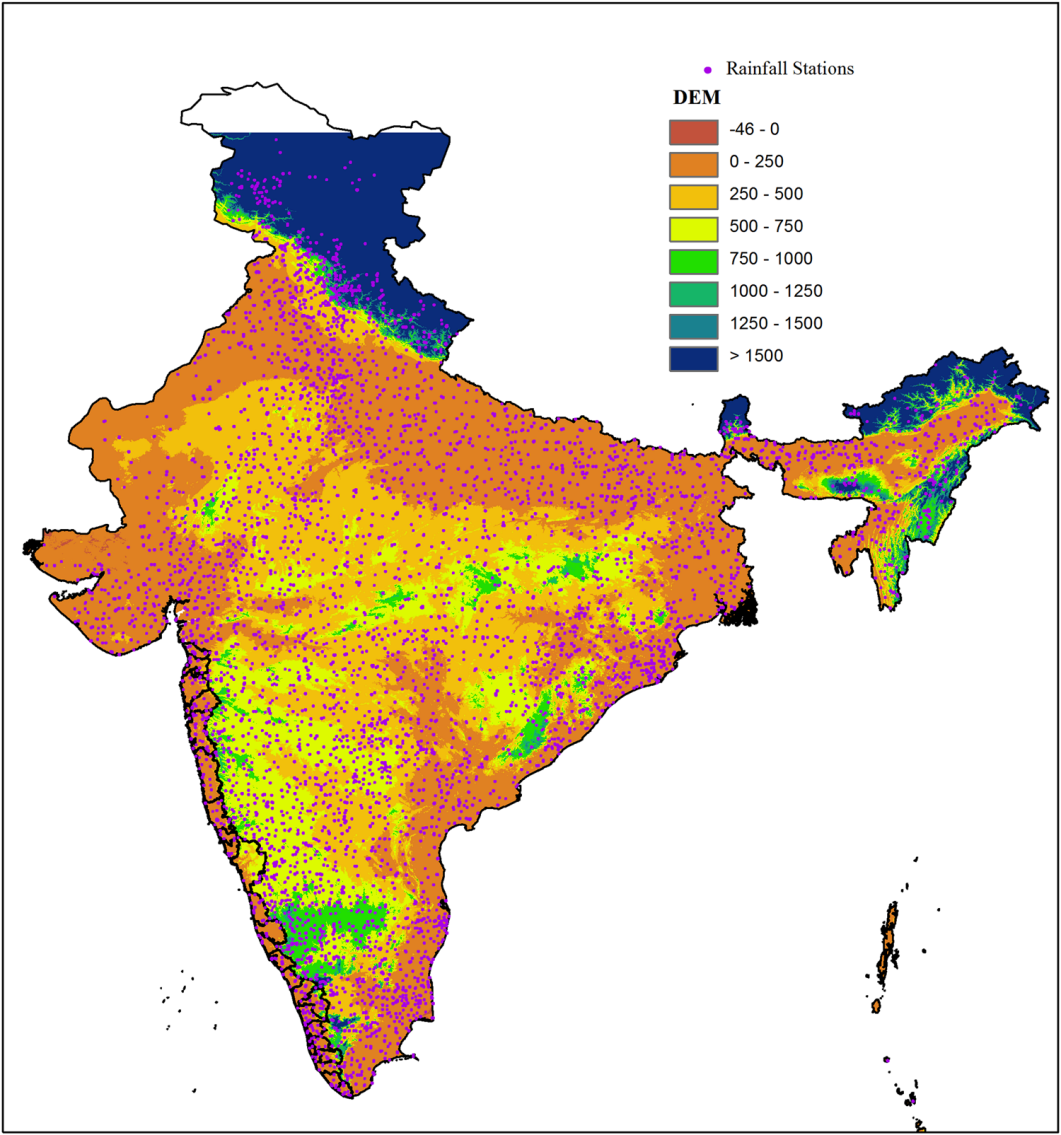
Topography of Indian land mass with gauge distribution.

TRMM Multi-satellite Precipitation Analysis (TMPA) 3B42 V7 and IMERG V5 daily *gauge calibrated* rainfall products have been used from 1998 to 2016. [TMPA data from 1998 to 2013 and IMERG is from 2014 to 2016]. Earlier studies show that TMPA and IMERG are in very good agreement over land^49^ as IMERG uses similar algorithm of TMPA. The details of the data sets used can be found from Thakur *et al*.^50^. India Meteorological Department (IMD), Govt. of India offers gridded rainfall data sets for the landmass of India, developed from the wide range of rain gauges. The data sets being used in the present study comprises with 0.25° × 0.25° spatial resolution and with daily temporal resolution. The basic rainfall data to develop these data sets are taken from the 6955 rain gauge stations over Indian land mass and at least 3000 rain gauges data have been used for any single day. The methodology followed to interpolate the data is Shepard interpolation technique^51^ and more details on the data sets can be found from^52^. The density of the rain gauges used in developing these data set along with the topography is shown in [Fig. 7]. While developing the data sets, directional effects have been taken care of but not the orographic corrections and the data sets do not include satellite measurements. These data sets are proven as the most reliable data sets over India and are being widely used for different applications such as validation of satellite products^53–55^, monsoon studies^56–58^ and extreme rainfall events^59–61^. For climatology of rainfall, same IMD rainfall product is used for the period 1961 to 1990. IMERG data set is re-sampled using interpolation technique from 0.1° × 0.1° to grid resolution of TMPA and IMD 0.25° × 0.25°.

Pair of daily Real-time Multivariate MJO series 1 (RMM1) and 2 (RMM2)^29^ were obtained from (http://www.bom.gov.au/climate/mjo/graphics/rmm). Meteorological parameters such as daily zonal (u) and meridional (v) component of wind at 200 mb, specific humidity and temperature profiles at different pressure level with grid resolution 2.5° × 2.5° were taken from NCEP/NCAR reanalysis^62^. These data sets have been used to estimate the daily tropical easterly Jet (TEJ) and moist static stability (MSS) using the formulae given below.

i $${\rm{TEJ}}={\rm{wind}}\,{\rm{at}}\,200\,{\rm{mb}}=\sqrt{(u2\,+v2)\,}\cdots $$

ii $${\rm{MSS}}=-\frac{\partial }{\partial p}(gz+{c}_{p}T+Lq)\cdots $$

where, g is acceleration due to gravity, z is height above the surface, c_p_ is specific heat at constant pressure, T is absolute air temperature, L is the latent heat of condensation, and q is specific humidity^26^. Surface Vertical velocity is derived from ERA- interim reanalysis 0.5° × 0.5° daily data set^63^. In addition Outgoing Longwave Radiation (OLR) is obtained from Very High Resolution Radiometer (VHRR) on board KALPANA-1 satellite^64^.

## Supplementary information

Supplementary Figures and Tables
