# Supplementary material for: A new perspective in understanding rainfall from satellites over a complex topographic region of India
Source: Sci Rep. 2019 Oct 30;9:15610. doi: 10.1038/s41598-019-52075-y (PMC6821882; doi:10.1038/s41598-019-52075-y)
Supplement: Supplementary file 1 — Supplementary Figures and Tables [file 41598_2019_52075_MOESM1_ESM.pdf]

A new perspective in understanding rainfall from satellites over a complex topographic region of India

Manoj Kumar Thakur[a,b], T.V. Lakshmi Kumar[a*], K. Koteswararao[c], Humberto Barbosa[d] and V. Brahmananda Rao[e]

[a]Atmospheric Science Research Laboratory, Department of Physics,
SRM Institute of Science and Technology, Kattankulathur, 603203, India
[b]Tribhuvan University, Kathmandu, Nepal
[c]Centre for Climate Change Research, Indian Institute of Tropical Meteorology, Pune, India
[d]Laboratorio de Analise e Processamento de Imagens de Satelites, Universiadade Federal, de Alogoas-UFAL,Brazil
[e]Instituto Nacional de Pesquisas Espaciais, INPE C.P. 515, São José dos Campos, SP, 12245-970, Brazil

lkumarap@hotmail.com

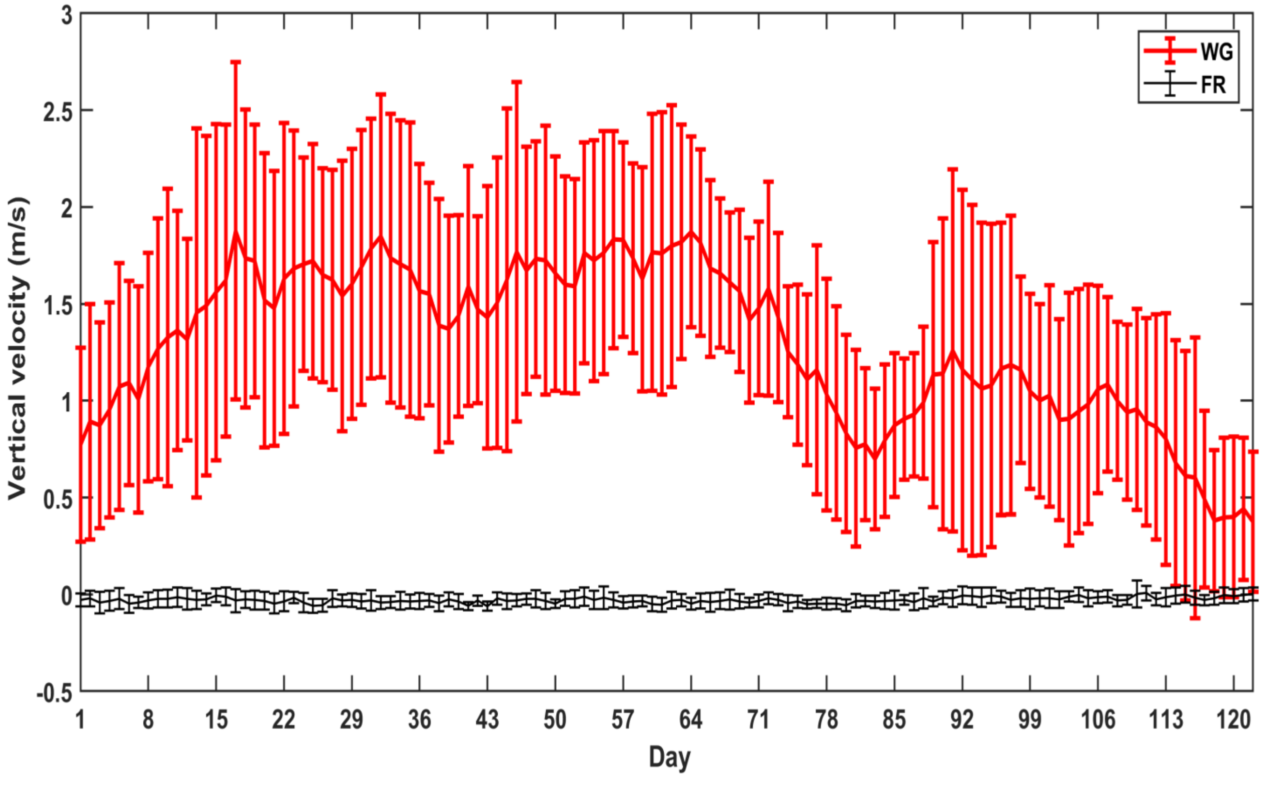

Fig.1 Time series plot of surface mean vertical velocity over Western Ghats (WG) and Flat region (FR) for selected grid box (0.5°) averaged from 2004 to 2015.

Table 1 Difference in Outgoing Longwave Radiation (OLR) in selected 0.5º grid box over Flat region (FR) and Western Ghats(WG) in different rainfall (RF /mm) range.

	Difference in OLR (W/m^2)		
Year	40≤RF<50	50≤RF<60	RF>60
2004	59.86	----	----
2009	46.20	56.04	59.49
2010	----	90.18	----
2014	52.03	70.16	34.58
2015	----	----	66.57
